# Reproducibility and Reuse of Adaptive Immune Receptor Repertoire Data

**DOI:** 10.3389/fimmu.2017.01418

**Published:** 2017-11-01

**Authors:** Felix Breden, Eline T. Luning Prak, Bjoern Peters, Florian Rubelt, Chaim A. Schramm, Christian E. Busse, Jason A. Vander Heiden, Scott Christley, Syed Ahmad Chan Bukhari, Adrian Thorogood, Frederick A. Matsen IV, Yariv Wine, Uri Laserson, David Klatzmann, Daniel C. Douek, Marie-Paule Lefranc, Andrew M. Collins, Tania Bubela, Steven H. Kleinstein, Corey T. Watson, Lindsay G. Cowell, Jamie K. Scott, Thomas B. Kepler

**Affiliations:** ^1^Department of Biological Sciences, Simon Fraser University, Burnaby, BC, Canada; ^2^Department of Pathology and Laboratory Medicine, Perelman School of Medicine, University of Pennsylvania, Philadelphia, PA, United States; ^3^La Jolla Institute for Allergy and Immunology, La Jolla, CA, United States; ^4^Department of Microbiology and Immunology, Institute for Immunity, Transplantation and Infection, Stanford University School of Medicine, Stanford, CA, United States; ^5^Vaccine Research Center, National Institute of Allergy and Infectious Diseases (NIAID), National Institutes of Health (NIH), Bethesda, MD, United States; ^6^Division of B Cell Immunology, Deutsches Krebsforschungszentrum (DKFZ), Heidelberg, Germany; ^7^Department of Neurology, Yale University School of Medicine, New Haven, CT, United States; ^8^Department of Clinical Sciences, University of Texas Southwestern Medical Center, Dallas, TX, United States; ^9^Department of Pathology, Yale University School of Medicine, New Haven, CT, United States; ^10^entre of Genomics and Policy, McGill University, Montreal, QC, Canada; ^11^Public Health Sciences Division and Computational Biology Program, Fred Hutchinson Cancer Research Center, Seattle, WA, United States; ^12^Department of Molecular Microbiology and Biotechnology, Tel Aviv University, Tel Aviv, Israel; ^13^Department of Genetics and Genome Sciences, Icahn School of Medicine at Mount Sinai, New York, NY, United States; ^14^Immunology-Immunopathology-Immunotherapy (i3 & i2B), Sorbonne Université, Paris, France; ^15^IMGT, LIGM, Institut de Génétique Humaine IGH, CNRS, University of Montpellier, Montpellier, France; ^16^School of Biotechnology and Biomolecular Sciences, University of New South Wales, Kensington, NSW, Australia; ^17^Faculty of Health Sciences, Simon Fraser University, Burnaby, BC, Canada; ^18^Department of Biochemistry and Molecular Genetics, University of Louisville School of Medicine, Louisville, KY, United States; ^19^Faculty of Health Sciences, Department of Molecular Biology and Biochemistry, Simon Fraser University, Burnaby, BC, Canada; ^20^Department of Microbiology, Boston University School of Medicine, Boston, MA, United States; ^21^Department of Mathematics and Statistics, Boston University, Boston, MA, United States

**Keywords:** B-cell receptors, T-cell receptors, data sharing, immunogenetics, community standards, high-throughput sequencing, immunoglobulins, antibodies

## Abstract

High-throughput sequencing (HTS) of immunoglobulin (B-cell receptor, antibody) and T-cell receptor repertoires has increased dramatically since the technique was introduced in 2009 (1–3). This experimental approach explores the maturation of the adaptive immune system and its response to antigens, pathogens, and disease conditions in exquisite detail. It holds significant promise for diagnostic and therapy-guiding applications. New technology often spreads rapidly, sometimes more rapidly than the understanding of how to make the products of that technology reliable, reproducible, or usable by others. As complex technologies have developed, scientific communities have come together to adopt common standards, protocols, and policies for generating and sharing data sets, such as the MIAME protocols developed for microarray experiments. The Adaptive Immune Receptor Repertoire (AIRR) Community formed in 2015 to address similar issues for HTS data of immune repertoires. The purpose of this perspective is to provide an overview of the AIRR Community’s founding principles and present the progress that the AIRR Community has made in developing standards of practice and data sharing protocols. Finally, and most important, we invite all interested parties to join this effort to facilitate sharing and use of these powerful data sets (join@airr-community.org).

## Introduction

The adaptive immune system provides protection against disease without inducing harmful autoimmunity; it reacts against the vast and ever-changing array of pathogens that an individual will encounter over a lifetime, while tolerating self. The variable regions of the adaptive immune receptors on B cells and T cells arise through the rearrangement of germline variable, diversity, and joining gene segments (4, 5). Humans each express over 100 million unique immunoglobulins (6) and a similar number of T-cell receptors (1, 7). The lymphocytes that express these receptors arise, proliferate, and die on time scales of hours to years (1, 8). Thus, the collection of B-cell and T-cell receptor variable region genes expressed at any given time—the adaptive immune receptor repertoire (AIRR)—is dynamic.

Immunoglobulin and T-cell receptor sequences have been studied for decades and several established databases exist including Kabat–Wu and Vbase2 (9, 10). Furthermore, there are databases that incorporate or allow viewing of structural data, such as IMGT, IEDB-3D, AntigenDB, and SAbDab [reviewed in Ref. (11)]. These data sets provide important insights into immune receptor–antigen interactions and can inform antibody engineering efforts. However, a single immunoglobulin or T-cell receptor sequence is but a drop of water in the ocean that is the immune repertoire. While many immune repertoire studies have been performed using a variety of methods [reviewed in Ref. (12)], adequate analysis of the repertoire as a whole was virtually impossible prior to the advent of high-throughput sequencing (HTS). Here, we focus on HTS-based profiling of AIRR.

Since HTS was first applied to AIRR profiling in 2009 (1, 3, 6, 7), there has been rapid advancement of both experimental and computational techniques. HTS of AIRRs (AIRR-seq) is yielding valuable insights into how variation in the AIRR differs across lymphocyte subsets (13–16) and anatomic compartments (17–20), varies over the course of a disease or with therapy (21–27), and is influenced by age (28–32), genetic background (33, 34), health status (19, 29, 35–37), antigen exposure (27, 38–40), and other factors. AIRR-seq data are increasingly important in the development of vaccines, monoclonal antibodies, cancer immunotherapies, and other applications [reviewed in Ref. (41)]. As the number of datasets continues to grow, comparative analyses of hundreds or even thousands of individuals will soon be feasible. Ensuring the reliability of such integrative analyses, however, will require the establishment of and adherence to standards for reporting and sharing data across multiple laboratories and centers.

## Challenges for Airr-Seq Data Sharing

Several challenges currently impede the effective sharing of AIRR-seq data. First, the storage and transport of such large datasets, which can comprise hundreds of millions of sequences (and hundreds of gigabytes) per study, require substantial time and resources. Second, deposition into public archives is not uniformly required by journals or funding agencies. As of September 4, 2017, a Wiki page on the B-T.CR forum^1^ lists 82 AIRR-seq studies that report full HTS data to a public archive,^2^ while 42 (34%) do not.^3^ Third, the information required to ensure appropriate use of such data by secondary users requires delineation (42). These challenges are not unique to AIRR-seq data. Indeed, the need for shared standards has been recognized and addressed for previous high-throughput technologies (43), including microarray data (44).

Another significant challenge for AIRR-seq data is that the processing pipeline between the experiment and the ultimate analysis of the data is lengthy and specialized (45–59). Beyond the steps required to process any HTS data, the annotation required of AIRR-seq data is unique to these genes and subject to substantial uncertainty (52). Unlike other genes, the antigen receptors of adaptive immunity are assembled through the recombination of randomly chosen gene segments, with non-templated nucleotides added to the junctions and nucleotides nibbled away from the gene segments (60). In B cells, somatic hypermutation during affinity maturation results in further diversification of immunoglobulin genes (61, 62). In order for these data to be effectively shared and reanalyzed, the development of new metadata standards specific to the experimental and bioinformatic methods associated with AIRR-seq are required.

## A Brief History of the AIRR Community

The AIRR Community was established in 2015 at a meeting organized by Felix Breden, Jamie Scott, and Thomas Kepler in Vancouver, BC, USA to address these data sharing challenges. Membership in the AIRR Community is open and is intended to cover all aspects of AIRR-seq technology and its uses. Membership includes researchers expert in the generation of AIRR data; statisticians and bioinformaticians versed in their analysis; informaticians and data security experts experienced in their management; basic scientists and physicians who turn to such data for critical insights; and experts in the ethical, legal, and policy implications of sharing AIRR data.

In 2015, the AIRR Community formed three Working Groups. The Minimal Standards Working Group was tasked with the development of a set of metadata standards for the publication and sharing of AIRR-seq datasets. The Tools and Resources Working Group is focused on the development of standardized resources to facilitate the comparison of AIRR-seq datasets and analysis tools, including collection, validation, and nomenclature of germline alleles. Finally, the Common Repository Working Group is working to establish requirements for repositories that will store AIRR data. The Working Groups are dynamic and often collaborate with each other, as methods evolve and applications of standards in one area (for example, metadata standards) impact other areas (data repository requirements). Full recommendations and membership lists for the Working Groups as well as video recordings of the 2016 AIRR Community meeting are available at http://www.airr-community.org. At the June 2016 meeting held at the National Institutes of Health (NIH), the AIRR Community ratified an initial set of recommendations that are summarized herein.

## Data Generation

Due to the complexity and diversity of the data sets being generated, the AIRR Community is developing best practices for the generation of AIRR-seq data. Such best practice guidelines will include, at a minimum: standard operating procedures for cell isolation and purification, including panels and gating strategies for flow cytometry; primers and protocols for amplification and sequencing of BCR or TCR rearrangements; and a clear description of library preparation and sequencing. Nomenclature is particularly important when it comes to the multiple stages of sample processing and data analysis. For example, what is meant by “raw data” differs among investigators, compounded by the fact that there are multiple levels of data preprocessing.

At present, the AIRR Community recommends that: (1) experimental protocols should be made available through a public repository granting digital object identifiers; (2) the change history of the experimental protocols, including details of what was changed and when the changes were made, should be made publicly available through the same repository; and (3) biological materials (e.g., plasmids, cell lines) should be made available to interested researchers *via* public repositories (e.g., Addgene for vectors, ATCC for cell lines), whenever possible.

## Data Sharing

For transparency and reliable reuse, experiments need to be sufficiently well annotated to allow evaluation of the quality of individual datasets and comparability of different datasets. Therefore, the AIRR Community has developed experimental metadata standards for AIRR-seq data generation, processing, and quality control. The data consist of the raw sequences and the processed sequences, while metadata include clinical and demographic data on study subjects and protocols for cell phenotyping, nucleic acid purification, AIRR amplicon production, HTS library preparation and sequencing, as well as documentation of the computational pipelines used to process the data. In publications or other forms of data sharing, these metadata sets and their components should be described in sufficient detail such that a person skilled in the art of AIRR sequencing and data analysis will be able to reproduce the experiment and data analyses that were performed. A manuscript describing a complete model for AIRR-seq data and metadata, and standardized terminology will soon be submitted.

Data sharing is also premised on the user’s ability to locate and access the data. The AIRR Community recommends that all published AIRR-seq data be deposited in designated public repositories that adhere to the AIRR Community minimal standards guidelines, namely, that the data should be made available under the least restrictive terms possible. Limited exceptions to respect commercial interests in intellectual property rights are under consideration by the AIRR Community. To facilitate data sharing, the AIRR Community is also establishing an AIRR Data Commons, comprised of multiple, distributed repositories optimized for storing and querying AIRR-seq data, and supported by a centralized Gateway. Under such an intermediate distributed model (43), interoperability and effective data sharing are ensured because participating repositories will be required to comply with the community-established data and metadata standards and certain technical requirements.

## Legal and Ethical Considerations

Adaptive immune receptor repertoire-seq data can be subject to regulations regarding informed consent, intellectual property, and ethical treatment of research subjects. During the process of making AIRR-seq data publicly available, researchers typically would attest that they have sought appropriate informed consent or other authorization for sharing, where applicable. To reduce the potential for a breach of privacy of research subjects, medical and demographic metadata should to be structured in such a way that individual research subjects are not identifiable. Access to health information is regulated by national and international laws, such as the Health Insurance Portability and Accountability Act in the United States or the EU Regulation 2016/679 in the European Union, which requires medical information and personal identifiers to be safeguarded. For studies using AIRR-seq data from human subjects, data must be collected following a protocol that has been approved by the researcher’s Institutional Review Board, which oversees human subjects’ protections and ensures that all studies are performed in a legal and ethical manner (63). Human subjects must provide informed consent, and there should be broad agreement in the consent language regarding the confidentiality of medical information and the use of AIRR-seq data and metadata for future research. Without such provisions, the data in the database may be too constrained, with respect to time or breadth of investigation, to be usable by investigators other than the initial data generators.

Whenever data or other items of potential commercial value are shared with others, the individuals who generated and deposited the data should to be given proper credit. Hence, users of the database should, at a minimum, credit the data depositors in any publication or grant application. One mechanism whereby these rules could be followed is to create an online form that must be completed before access to the database is granted. Such a form would essentially be a contract for using the data. Enforcement of the terms of the contract could include monitoring of data use and denying access to the database should the terms of the data-use agreement be violated.

To facilitate broad access to and use of AIRR-seq data, the data should be made available under the least-restrictive terms possible. The default data sharing policy should be to deposit data in a public domain database with no restrictions over deposit, access, storage, curation, or use. For data deposited in public domain databases/repositories, neither the depositors nor the repositories should be permitted to interfere with access to and use of the data by others, including through the assertion of any intellectual property rights. Exceptions to open data sharing may arise in circumstances in which open data sharing would come into conflict with the law, such as those pertaining to personal privacy and protected health information, or into conflict with decisions made by an Institutional Review Board.

## Data Analysis

The AIRR Community strongly advocates the use of statistical methods for data analysis and hypothesis testing. Statistical methods systematically characterize error, quantify uncertainty, and provide a measure of confidence for inferences. Statistical methods also form the basis for data analysis in all other realms of biomedical and scientific research and should be adopted for AIRR-seq data. Expanded production of AIRR-seq data has been supported by a proliferation of computational tools for their processing and analysis, including tools for variable region gene annotation, inference of clonal history and partitioning and visualization (16, 46–52, 55, 56, 64–69). To encourage broad and well-informed use of these tools, the AIRR Community recommends that analysis software be released under an Open Source Initiative approved license, hosted on a publicly available website or repository with versioning, and be designed for modularity and inter-operability with other software. The AIRR Community will promote best practices in AIRR-seq data analysis by: (1) developing and publishing common criteria for the evaluation of statistical methods; (2) providing common “gold-standard” datasets of multiple types for use in software development, testing, and calibration; and (3) establishing best practices for data sharing and analysis software platforms.

## Conclusion

Members of the AIRR Community have worked together for over 2 years with enthusiasm, driven by the belief that optimizing the reproducibility and sharing of AIRR-seq data will have a profound and positive effect on biomedical research and patient care. To encourage widespread adoption, the AIRR Community recommends that journals and funding agencies require AIRR-seq data be made available through a public data repository after publication or as negotiated in data-sharing agreements for unpublished data. The success of this initiative is also critically dependent upon acceptance by the researchers who generate and use AIRR-seq data. While members of the AIRR Community have tried to be inclusive through developing contacts with researchers in the field and extensively advertising the annual meetings, there are likely to be many researchers who generate, analyze, and use AIRR-seq data, who are not aware of the AIRR Community initiative. Community “buy in” results from creating data standards that are transparently developed through public discussion, robustly evaluated, and periodically updated as the field advances. This Perspective represents an open invitation to the larger scientific community to participate in and adopt the AIRR initiative. To that end, we welcome feedback on this Perspective and on the AIRR Community’s efforts to date. Individuals interested in working on any facet of this important initiative are invited to attend, in person or online, the 2017 Community Meeting hosted by the NIH in Rockville, MD, USA, December 3–6, 2017. Most of all, we encourage anyone who is interested to join the AIRR Community^4^ and participate in the working groups.

## Author Contributions

FB, EP, JS, and TK conceived of and wrote the manuscript. All other authors contributed ideas and/or proposed revisions to the text. The AIRR Community Working Groups developed and wrote the recommendations described herein.

## Conflict of Interest Statement

The authors declare that the research was conducted in the absence of any commercial or financial relationships that could be construed as a potential conflict of interest.

## Footnotes

^1^https://www.b-t.cr.

^2^https://www.b-t.cr/t/317.

^3^https://www.b-t.cr/t/426.

^4^join@airr-community.org.

## References

[1] Freeman JD Warren RL Webb JR Nelson BH Holt RA. Profiling the T-cell receptor beta-chain repertoire by massively parallel sequencing. Genome Res (2009) 19(10):1817–24. 10.1101/gr.092924.109 19541912 PMC2765271

[2] Robins HS Campregher PV Srivastava SK Wacher A Turtle CJ Kahsai O Comprehensive assessment of T-cell receptor beta-chain diversity in alphabeta T cells. Blood (2009) 114(19):4099–107. 10.1182/blood-2009-04-217604 19706884 PMC2774550

[3] Weinstein JA Jiang N White RA III Fisher DS Quake SR. High-throughput sequencing of the zebrafish antibody repertoire. Science (2009) 324(5928):807–10. 10.1126/science.1170020 19423829 PMC3086368

[4] Sakano H Maki R Kurosawa Y Roeder W Tonegawa S. Two types of somatic recombination are necessary for the generation of complete immunoglobulin heavy-chain genes. Nature (1980) 286(5774):676–83. 10.1038/286676a0 6774258

[5] Sakano H Kurosawa Y Weigert M Tonegawa S. Identification and nucleotide sequence of a diversity DNA segment (D) of immunoglobulin heavy-chain genes. Nature (1981) 290(5807):562–5. 10.1038/290562a0 6783961

[6] Glanville J Zhai W Berka J Telman D Huerta G Mehta GR Precise determination of the diversity of a combinatorial antibody library gives insight into the human immunoglobulin repertoire. Proc Natl Acad Sci U S A (2009) 106(48):20216–21. 10.1073/pnas.0909775106 19875695 PMC2787155

[7] Robins H. Immunosequencing: applications of immune repertoire deep sequencing. Curr Opin Immunol (2013) 25(5):646–52. 10.1016/j.coi.2013.09.017 24140071

[8] McLean AR Michie CA. In vivo estimates of division and death rates of human T lymphocytes. Proc Natl Acad Sci U S A (1995) 92(9):3707–11. 10.1073/pnas.92.9.3707 7731969 PMC42030

[9] Wu TT Kabat EA. An analysis of the sequences of the variable regions of Bence Jones proteins and myeloma light chains and their implications for antibody complementarity. J Exp Med (1970) 132(2):211–50. 10.1084/jem.132.2.211 5508247 PMC2138737

[10] Retter I Althaus HH Münch R Müller W. VBASE2, an integrative V gene database. Nucleic Acids Res (2005) 33(Database issue):D671–4. 10.1093/nar/gki088 15608286 PMC540042

[11] Dunbar J Krawczyk K Leem J Baker T Fuchs A Georges G SAbDab: the structural antibody database. Nucleic Acids Res (2014) 42(Database issue):D1140–6. 10.1093/nar/gkt1043 24214988 PMC3965125

[12] Six A Mariotti-Ferrandiz ME Chaara W Magadan S Pham HP Lefranc MP The past, present, and future of immune repertoire biology – the rise of next-generation repertoire analysis. Front Immunol (2013) 4:413. 10.3389/fimmu.2013.00413 24348479 PMC3841818

[13] Mroczek ES Ippolito GC Rogosch T Hoi KH Hwangpo TA Brand MG Differences in the composition of the human antibody repertoire by B cell subsets in the blood. Front Immunol (2014) 5:96. 10.3389/fimmu.2014.00096 24678310 PMC3958703

[14] Wu YC Kipling D Leong HS Martin V Ademokun AA Dunn-Walters DK. High-throughput immunoglobulin repertoire analysis distinguishes between human IgM memory and switched memory B-cell populations. Blood (2010) 116(7):1070–8. 10.1182/blood-2010-03-275859 20457872 PMC2938129

[15] Martin VG Wu YB Townsend CL Lu GH O’Hare JS Mozeika A Transitional B cells in early human B cell development – time to revisit the paradigm? Front Immunol (2016) 7:546. 10.3389/fimmu.2016.00546 27994589 PMC5133252

[16] Bashford-Rogers RJ Palser AL Huntly BJ Rance R Vassiliou GS Follows GA Network properties derived from deep sequencing of human B-cell receptor repertoires delineate B-cell populations. Genome Res (2013) 23(11):1874–84. 10.1101/gr.154815.113 23742949 PMC3814887

[17] Briney BS Willis JR Finn JA McKinney BA Crowe JE Jr. Tissue-specific expressed antibody variable gene repertoires. PLoS One (2014) 9(6):e100839. 10.1371/journal.pone.0100839 24956460 PMC4067404

[18] Meng W Zhang B Schwartz GW Rosenfeld AM Ren D Thome JJC An atlas of B-cell clonal distribution in the human body. Nat Biotechnol (2017) 35(9):879–84. 10.1038/nbt.3942 28829438 PMC5679700

[19] Stern JN Yaari G Vander Heiden JA Church G Donahue WF Hintzen RQ B cells populating the multiple sclerosis brain mature in the draining cervical lymph nodes. Sci Transl Med (2014) 6(248):248ra107. 10.1126/scitranslmed.3008879 25100741 PMC4388137

[20] Sathaliyawala T Kubota M Yudanin N Turner D Camp P Thome JJ Distribution and compartmentalization of human circulating and tissue-resident memory T cell subsets. Immunity (2013) 38(1):187–97. 10.1016/j.immuni.2012.09.020 23260195 PMC3557604

[21] Heather JM Best K Oakes T Gray ER Roe JK Thomas N Dynamic perturbations of the T-cell receptor repertoire in chronic HIV infection and following antiretroviral therapy. Front Immunol (2015) 6:644 10.3389/fimmu.2015.00644 26793190 PMC4707277

[22] Racanelli V Brunetti C De Re V Caggiari L De Zorzi M Leone P Antibody V(h) repertoire differences between resolving and chronically evolving hepatitis C virus infections. PLoS One (2011) 6(9):e25606. 10.1371/journal.pone.0025606 21980500 PMC3182224

[23] Faham M Zheng J Moorhead M Carlton VE Stow P Coustan-Smith E Deep-sequencing approach for minimal residual disease detection in acute lymphoblastic leukemia. Blood (2012) 120(26):5173–80. 10.1182/blood-2012-07-444042 23074282 PMC3537310

[24] Weng WK Armstrong R Arai S Desmarais C Hoppe R Kim YH. Minimal residual disease monitoring with high-throughput sequencing of T cell receptors in cutaneous T cell lymphoma. Sci Transl Med (2013) 5(214):214ra171. 10.1126/scitranslmed.3007420 24307695

[25] Kalos M Levine BL Porter DL Katz S Grupp SA Bagg A T cells with chimeric antigen receptors have potent antitumor effects and can establish memory in patients with advanced leukemia. Sci Transl Med (2011) 3(95):95ra73. 10.1126/scitranslmed.3002842 21832238 PMC3393096

[26] Morris H DeWolf S Robins H Sprangers B LoCascio SA Shonts BA Tracking donor-reactive T cells: evidence for clonal deletion in tolerant kidney transplant patients. Sci Transl Med (2015) 7(272):272ra10 10.1126/scitranslmed.3010760 PMC4360892 25632034

[27] Havenar-Daughton C Carnathan DG Torrents de la Peña A Pauthner M Briney B Reiss SM Direct probing of germinal center responses reveals immunological features and bottlenecks for neutralizing antibody responses to HIV env trimer. Cell Rep (2016) 17(9):2195–209. 10.1016/j.celrep.2016.10.085 27880897 PMC5142765

[28] Russell Knode LM Naradikian MS Myles A Scholz JL Hao Y Liu D Age-associated B cells express a diverse repertoire of VH and Vkappa genes with somatic hypermutation. J Immunol (2017) 198(5):1921–7. 10.4049/jimmunol.1601106 28093524 PMC5322232

[29] Gibson KL Wu YC Barnett Y Duggan O Vaughan R Kondeatis E B-cell diversity decreases in old age and is correlated with poor health status. Aging Cell (2009) 8(1):18–25. 10.1111/j.1474-9726.2008.00443.x 18986373 PMC2667647

[30] Qi Q Liu Y Cheng Y Glanville J Zhang D Lee JY Diversity and clonal selection in the human T-cell repertoire. Proc Natl Acad Sci U S A (2014) 111(36):13139–44. 10.1073/pnas.1409155111 25157137 PMC4246948

[31] Rechavi E Lev A Lee YN Simon AJ Yinon Y Lipitz S Timely and spatially regulated maturation of B and T cell repertoire during human fetal development. Sci Transl Med (2015) 7(276):276ra25 10.1126/scitranslmed.aaa0072 25717098

[32] Guo C Wang Q Cao X Yang Y Liu X An L High-throughput sequencing reveals immunological characteristics of the TRB-/IgH-CDR3 region of umbilical cord blood. J Pediatr (2016) 176:69–78.e1. 10.1016/j.jpeds.2016.05.078 27373756

[33] Notarangelo LD Kim MS Walter JE Lee YN. Human RAG mutations: biochemistry and clinical implications. Nat Rev Immunol (2016) 16(4):234–46. 10.1038/nri.2016.28 26996199 PMC5757527

[34] Watson CT Steinberg KM Huddleston J Warren RL Malig M Schein J Complete haplotype sequence of the human immunoglobulin heavy-chain variable, diversity, and joining genes and characterization of allelic and copy-number variation. Am J Hum Genet (2013) 92(4):530–46. 10.1016/j.ajhg.2013.03.004 23541343 PMC3617388

[35] Tipton CM Fucile CF Darce J Chida A Ichikawa T Gregoretti I Diversity, cellular origin and autoreactivity of antibody-secreting cell population expansions in acute systemic lupus erythematosus. Nat Immunol (2015) 16(7):755–65. 10.1038/ni.3175 26006014 PMC4512288

[36] Stamatopoulos K Belessi C Moreno C Boudjograh M Guida G Smilevska T Over 20% of patients with chronic lymphocytic leukemia carry stereotyped receptors: pathogenetic implications and clinical correlations. Blood (2007) 109(1):259–70. 10.1182/blood-2006-03-012948 16985177

[37] Rubelt F Bolen CR McGuire HM Vander Heiden JA Gadala-Maria D Levin M Individual heritable differences result in unique cell lymphocyte receptor repertoires of naive and antigen-experienced cells. Nat Commun (2016) 7:11112 10.1038/ncomms11112 27005435 PMC5191574

[38] Laserson U Vigneault F Gadala-Maria D Yaari G Uduman M Vander Heiden JA High-resolution antibody dynamics of vaccine-induced immune responses. Proc Natl Acad Sci U S A (2014) 111(13):4928–33. 10.1073/pnas.1323862111 24639495 PMC3977259

[39] Vollmers C Sit RV Weinstein JA Dekker CL Quake SR. Genetic measurement of memory B-cell recall using antibody repertoire sequencing. Proc Natl Acad Sci U S A (2013) 110(33):13463–8. 10.1073/pnas.1312146110 23898164 PMC3746854

[40] Lavinder JJ Wine Y Giesecke C Ippolito GC Horton AP Lungu OI Identification and characterization of the constituent human serum antibodies elicited by vaccination. Proc Natl Acad Sci U S A (2014) 111(6):2259–64. 10.1073/pnas.1317793111 24469811 PMC3926051

[41] Georgiou G Ippolito GC Beausang J Busse CE Wardemann H Quake SR. The promise and challenge of high-throughput sequencing of the antibody repertoire. Nat Biotechnol (2014) 32(2):158–68. 10.1038/nbt.2782 24441474 PMC4113560

[42] Bhattacharya S Andorf S Gomes L Dunn P Schaefer H Pontius J ImmPort: disseminating data to the public for the future of immunology. Immunol Res (2014) 58(2–3):234–9. 10.1007/s12026-014-8516-1 24791905

[43] Contreras JL Reichman JH DATA ACCESS. Sharing by design: data and decentralized commons. Science (2015) 350(6266):1312–4. 10.1126/science.aaa7485 26659039 PMC4811371

[44] Brazma A Hingamp P Quackenbush J Sherlock G Spellman P Stoeckert C Minimum information about a microarray experiment (MIAME)-toward standards for microarray data. Nat Genet (2001) 29(4):365–71. 10.1038/ng1201-365 11726920

[45] Yaari G Kleinstein SH. Practical guidelines for B-cell receptor repertoire sequencing analysis. Genome Med (2015) 7:121. 10.1186/s13073-015-0243-2 26589402 PMC4654805

[46] Imkeller K Arndt PF Wardemann H Busse CE. sciReptor: analysis of single-cell level immunoglobulin repertoires. BMC Bioinformatics (2016) 17:67. 10.1186/s12859-016-0920-1 26847109 PMC4743164

[47] Rosenfeld AM Meng W Luning Prak ET Hershberg U. ImmuneDB: a system for the analysis and exploration of high-throughput adaptive immune receptor sequencing data. Bioinformatics (2017) 33(2):292–3. 10.1093/bioinformatics/btw593 27616708 PMC5254080

[48] Rogosch T Kerzel S Hoi KH Zhang Z Maier RF Ippolito GC Immunoglobulin analysis tool: a novel tool for the analysis of human and mouse heavy and light chain transcripts. Front Immunol (2012) 3:176. 10.3389/fimmu.2012.00176 22754554 PMC3384897

[49] Ralph DK Matsen FA IV. Likelihood-based inference of B cell clonal families. PLoS Comput Biol (2016) 12(10):e1005086. 10.1371/journal.pcbi.1005086 27749910 PMC5066976

[50] Bolotin DA Poslavsky S Mitrophanov I Shugay M Mamedov IZ Putintseva EV MiXCR: software for comprehensive adaptive immunity profiling. Nat Methods (2015) 12(5):380–1. 10.1038/nmeth.3364 25924071

[51] Shugay M Bagaev DV Turchaninova MA Bolotin DA Britanova OV Putintseva EV VDJtools: unifying post-analysis of T cell receptor repertoires. PLoS Comput Biol (2015) 11(11):e1004503. 10.1371/journal.pcbi.1004503 26606115 PMC4659587

[52] Kepler TB. Reconstructing a B-cell clonal lineage. I. Statistical inference of unobserved ancestors. F1000Res (2013) 2:103. 10.12688/f1000research.2-103.v1 24555054 PMC3901458

[53] Kepler TB Munshaw S Wiehe K Zhang R Yu JS Woods CW Reconstructing a B-cell clonal lineage. II. Mutation, selection, and affinity maturation. Front Immunol (2014) 5:170. 10.3389/fimmu.2014.00170 24795717 PMC4001017

[54] Liberman G Benichou JI Maman Y Glanville J Alter I Louzoun Y. Estimate of within population incremental selection through branch imbalance in lineage trees. Nucleic Acids Res (2016) 44(5):e46. 10.1093/nar/gkv1198 26586802 PMC4797263

[55] Vincent B iWAS – a novel approach to analyzing next generation sequence data for immunology. Cell Immunol (2016) 299:6–13. 10.1016/j.cellimm.2015.10.012 26547365 PMC4698203

[56] Volpe JM Cowell LG Kepler TB SoDA: implementation of a 3D alignment algorithm for inference of antigen receptor recombinations. Bioinformatics (2006) 22(4):438–44. 10.1093/bioinformatics/btk004 16357034

[57] Alamyar E Duroux P Lefranc MP Giudicelli V IMGT((R)) tools for the nucleotide analysis of immunoglobulin (IG) and T cell receptor (TR) V-(D)-J repertoires, polymorphisms, and IG mutations: IMGT/V-QUEST and IMGT/HighV-QUEST for NGS. Methods Mol Biol (2012) 882:569–604. 10.1007/978-1-61779-842-9_32 22665256

[58] Ye J Ma N Madden TL Ostell JM IgBLAST: an immunoglobulin variable domain sequence analysis tool. Nucleic Acids Res (2013) 41(Web Server issue):W34–40. 10.1093/nar/gkt382 23671333 PMC3692102

[59] Zhang B Meng W Luning Prak ET Hershberg U. Discrimination of germline V genes at different sequencing lengths and mutational burdens: a new tool for identifying and evaluating the reliability of V gene assignment. J Immunol Methods (2015) 427:105–16. 10.1016/j.jim.2015.10.009 26529062 PMC4811607

[60] Gellert M. V(D)J recombination: RAG proteins, repair factors, and regulation. Annu Rev Biochem (2002) 71:101–32. 10.1146/annurev.biochem.71.090501.150203 12045092

[61] Weigert MG Cesari IM Yonkovich SJ Cohn M Variability in the lambda light chain sequences of mouse antibody. Nature (1970) 228(5276):1045–7. 10.1038/2281045a0 5483159

[62] Jacob J Kelsoe G Rajewsky K Weiss U. Intraclonal generation of antibody mutants in germinal centres. Nature (1991) 354(6352):389–92. 10.1038/354389a0 1956400

[63] Freedman RS Cantor SB Merriman KW Edgerton ME. 2013 HIPAA changes provide opportunities and challenges for researchers: perspectives from a cancer center. Clin Cancer Res (2016) 22(3):533–9. 10.1158/1078-0432.CCR-15-2155 26832744

[64] Gupta NT Adams KD Briggs AW Timberlake SC Vigneault F Kleinstein SH. Hierarchical clustering can identify B cell clones with high confidence in ig repertoire sequencing data. J Immunol (2017) 198(6):2489–99. 10.4049/jimmunol.1601850 28179494 PMC5340603

[65] Gupta NT Vander Heiden JA Uduman M Gadala-Maria D Yaari G Kleinstein SH. Change-O: a toolkit for analyzing large-scale B cell immunoglobulin repertoire sequencing data. Bioinformatics (2015) 31(20):3356–8. 10.1093/bioinformatics/btv359 26069265 PMC4793929

[66] Vander Heiden JA Yaari G Uduman M Stern JN O’Connor KC Hafler DA pRESTO: a toolkit for processing high-throughput sequencing raw reads of lymphocyte receptor repertoires. Bioinformatics (2014) 30(13):1930–2. 10.1093/bioinformatics/btu138 24618469 PMC4071206

[67] Ostmeyer J Christley S Rounds WH Toby I Greenberg BM Monson NL Statistical classifiers for diagnosing disease from immune repertoires: a case study using multiple sclerosis. BMC Bioinformatics (2017) 18(1):401. 10.1186/s12859-017-1814-6 28882107 PMC5588725

[68] Toby IT Levin MK Salinas EA Christley S Bhattacharya S Breden F VDJML: a file format with tools for capturing the results of inferring immune receptor rearrangements. BMC Bioinformatics (2016) 17(Suppl 13):333. 10.1186/s12859-016-1214-3 27766961 PMC5073965

[69] Christley S Levin MK Toby I Fonner J Monson N Rounds WH VDJPipe: a pipelined tool for pre-processing immune repertoire sequencing data. BMC Bioinformatics (2017) 18(1):448 10.1186/s12859-017-1853-z 29020925 PMC5637252

